# Supplementary material for: The Impact of City-Led Neighborhood Action on the Coproduction of Neighborhood Quality and Safety in Buffalo, NY
Source: Int J Environ Res Public Health. 2025 Feb 26;22(3):341. doi: 10.3390/ijerph22030341 (PMC11942396; doi:10.3390/ijerph22030341)
Supplement: Supplementary file 1 [file ijerph-22-00341-s001.zip › ijerph-3412440-supplementary.pdf]

Supplemental Material

Detail on the data sources, variables, study design, analytical strategy, matching, effect estimation, and full quantitative results can be found in Dickens et al. [8]. We provide a summary here.

The City of Buffalo's Open Data Portal contains administrative city data on each property in the city. These data include police incidents, building permits, land use, housing code violations, assessed value, and more. The city also maintains data on where Clean Sweeps occurred between 2009 and 2022. For each year of the Clean Sweep, we drew comparable clusters of properties that had not received a Clean Sweep using property-level data such that the treated and untreated clusters were statistically analogous. We then simulated a matched pair randomized controlled trial using frontier matching. In this way, we were able to compare the risk ratio of reporting of crime incidents and service needs between properties that received a Clean Sweep and those that did not.

Outcome variables were constructed using panel data on police incidents (911 calls and police-reported incidents) and calls for service (311 calls). 911 data was categorized as "violent," "property," "disorderly (drug)," and "all other offenses" using the FBI's uniform crime reporting program (UCR). We grouped 311 subtypes into two categories: call types related to physical disorder (blight) that are directly and visibly addressed during the Clean Sweep, and those that are not. The follow-up time horizon for the outcome variables is the same for each set of matched treated and untreated properties. For police incidents, the start date is the day after the Clean Sweep. For service incidents, as city staff often input 311 requests after a Clean Sweep on behalf of residents, the start date is five days after the intervention. The time horizon for the endline was 6 months, as after 6 months a new Clean Sweep season begins and may influence results from the previous year. Dummy variables were generated with 1 indicating the event (911 or 311 call) had occurred and 0 if the event had not occurred in the timeframe.

Table S1: Risk ratios for 911 and 311 reporting at properties that received a Clean Sweep compared to those that did not, 3-month follow-up

	Marginal probability		Risk ratio	95% Confidence interval
	Untreated	Treated		
All police incident types	.0645	.0632	0.979	(0.921, 1.042)
Violent crime	.0038	.004	1.060	(0.816, 1.376)
Property crime	.0244	.0228	0.936	(0.848, 1.033)
Disorderly (drug) crime	.0047	.0062	1.321	(1.034, 1.687)
Other crime	.0395	.0393	0.995	(0.913, 1.085)
All service incident types	.1286	.1343	1.044	(0.993, 1.097)
Clean Sweep related incidents	.0401	.0437	1.090	(1.007, 1.180)
Non-Clean Sweep related incidents	.0989	.1025	1.037	(0.978, 1.099)

*Coefficients presented are risk ratio estimates for receiving treatment from multivariate logistic regression models averaged across all 11 years. *** p < 0.001, **p<0.01, *p<0.05.*

Table S2: Risk ratios for 911 and 311 reporting at properties that received a Clean Sweep compared to those that did not, 6-month follow-up

	Marginal probability		Risk ratio	95% Confidence interval
	Untreated	Treated		
All police incident types	.0978	.0965	0.986	(0.939, 1.036)
Violent crime	.0066	.0067	1.021	(0.834, 1.249)
Property crime	.0361	.0335	0.927	(0.858, 1.002)
Disorderly (drug) crime	.0077	.0109	1.415***	(1.162, 1.723)
Other crime	.0641	.0634	0.989	(0.926, 1.056)
All service incident types	.2019	.205	1.015	(0.975, 1.058)
Clean-Sweep related incidents	.061	.0664	1.088*	(1.017, 1.164)
Non-Clean-Sweep related incidents	.1617	.1626	1.006	(0.959, 1.055)

*Coefficients presented are risk ratio estimates for receiving treatment from multivariate logistic regression models averaged across all 11 years. *** p < 0.001, *p<0.05*
